# Do consumers continue to use O2O food delivery services in the post-pandemic era? Roles of sedentary lifestyle

**DOI:** 10.1016/j.heliyon.2023.e19131

**Published:** 2023-08-15

**Authors:** Pinyi Yao, Syuhaily Osman, Mohamad Fazli Sabri, Norzalina Zainudin, Yezheng Li

**Affiliations:** a Faculty of Human Ecology, Universiti Putra Malaysia, Serdang 43400, Malaysia; b Business School, Guilin University of Technology, Guilin 541004, China

**Keywords:** O2O food delivery, Post-COVID-19, UTAUT2, Network externalities, Sedentary lifestyle

## Abstract

Online-to-offline food delivery (O2OFD) services have become popular worldwide, and consumers' O2OFD usage and sedentary behavior have increased during the COVID-19 pandemic. However, whether consumers will continue to use O2OFD in the post-pandemic era is uncertain, and the relationship between sedentary lifestyle and O2OFD usage is poorly understood to date. Therefore, this study aims to investigate consumers' continued intention to use O2OFD in the post-pandemic era and their subsequent use behavior, as well as to explore the roles of sedentary lifestyle. A research model based on the unified theory of acceptance and use of technology 2 (UTAUT2), integrating network externalities and including sedentary lifestyle, was proposed. A two-stage online survey was conducted in China, with 409 eligible responses used in the data analysis. The results indicate that habit, perceived network size, and perceived complementarity were the main predictors of continued intention, whereas habit and continued intention were the primary determinants of use behavior. Sedentary lifestyle was positively related to O2OFD use behavior and moderated the effects of habit and continued intention on use behavior. In addition to its theoretical contributions, this study has implications for public policies, marketing strategies, and consumer well-being.

## 1 Introduction

Food delivery is a popular and typical example of online-to-offline (O2O) commerce that connects online channels with offline business activities [1,2]. Food delivery services offer consumers an alternative option to cooking at home or dining out [3,4], where they can purchase food via online channels (usually mobile applications) and receive delivery at the doorstep of their house or workplace. Such food delivery links the convenience of online services with essential offline food consumption and is therefore called online-to-offline food delivery (O2OFD). Numerous local restaurants or food outlets usually work with third-party platforms (i.e., O2OFD platforms), of which some franchises (e.g., McDonald's and KFC) may also establish their own platforms, to provide the ready-to-eat food (often referred to as "take-away" or "take-out" food) to neighborhood diners. O2OFD platforms, such as Meituan and Ele.me in China, Swiggy and Zomato in India, Grubhub and Doordash in the USA, and Grabfood in Southeast Asia, are generally responsible for operating online applications and providing instant delivery services.

Although the O2OFD industry has been expanding since its emergence, it remained lukewarm worldwide [5] until the outbreak of the coronavirus disease 2019 (COVID-19). In the early phases of the pandemic, many countries around the world implemented restrictive measures, such as the stay-at-home order, to prevent the spread of COVID-19. Due to the social distancing policy or fear of COVID-19, many people actively or passively reduced the frequency of going out for groceries or meals, causing growing numbers of people to start or increase their use of O2OFD services. As reported, restaurant food delivery worldwide increased by approximately 50% in 2020 over 2019 due to the COVID-19 pandemic [6], and over 1.6 billion people globally used some form of O2OFD service in 2021 [7]. In addition to the increase in user size, many traditional offline restaurants, such as luxury hotel restaurants [8], started to offer O2OFD services to survive in times of crisis. Additionally, new services related to the O2OFD emerged or became popular during the pandemic. For example, fresh food and medicine delivery had become common in China [9], drone delivery was becoming popular in South Korea [10], and innovative services called Home Chef and DIY meal kits were appearing in Spain [11]. In brief, the pandemic has activated and reshaped the O2OFD industry.

As China announced in December 2022 the easing of its COVID-19 management [12], which was regarded as one of the world's tightest COVID-19 policies that had shielded China's 1.4 billion people from the virus but also disrupted their daily lives to some extent, it can be said that the post-pandemic era has indeed begun. In May 2023, the World Health Organization (WHO) declared that the COVID-19 pandemic no longer constitutes a public health emergency of international concern [13], signaling that the world will resume normal operations as before. In terms of the O2OFD market in the post-pandemic era, as COVID-related restrictions are gradually lifted, the main problem is whether consumers will continue to use O2OFD services as frequently as they did during the pandemic remains uncertain. Nevertheless, it is worth acknowledging that food delivery will become the new normal for restaurants and diners around the world in the foreseeable future [14]. Accordingly, it is necessary to continue studying relevant consumer behavior in the post-pandemic era, which can not only enable O2OFD platforms and various restaurants to remain resilient in their business but also help consumers make more informed decisions.

Many studies have focused on consumer behavior regarding the use of O2OFD services during the pandemic (e.g., Refs. [[15], [16], [17], [18]]). A thematic review found that factors influencing consumers' use of O2OFD services during the pandemic involved eight major aspects in addition to COVID-related factors: (1) utilitarian aspect, (2) system design aspect, (3) hedonic aspect, (4) individual characteristic aspect, (5) service aspect, (6) risk aspect, (7) social aspect, and (8) food aspect [1]. These findings are similar to the previous literature on e-commerce or information systems. However, consumers' purchasing behavior during the pandemic may be unusual [19], and their expectations during a crisis were not usual expectations [20], resulting that consumers' behavior may change again in the post-pandemic era. In addition, new changes arising from the pandemic, such as the increase in user size and the emergence of new services, may encourage consumers to continue using O2OFD services. However, consumer behavior regarding the use of O2OFD services in the post-pandemic era has not yet been investigated. Therefore, this study aims to investigate the factors that influence consumers' continued use of O2OFD services in the post-pandemic era.

Similar to other O2O businesses, O2OFD can be seen by users as an innovative information system service, and its popularity cannot be achieved without the development of information technology, especially mobile Internet technology [21]. Consequently, theories of technology acceptance and use have become one of the dominant theoretical lenses through which researchers understand consumer behavior in using O2OFD services. The unified theory of acceptance and use of technology 2 (UTAUT2) is regarded as the theory that best matches O2O commerce [2], and many O2OFD-related studies have adopted it to explain consumer behavior (e.g., Refs. [[22], [23], [24], [25]]). However, previous studies have usually replicated all the original UTAUT2 predictors rather than modifying the original model appropriately according to the study purpose, resulting in overly large models or relatively superficial discussions. Furthermore, previous studies, including those using other theories, have tended to focus on understanding the determinants of consumer intention rather than behavior [1]. This is because attitude-related theories from social psychology, such as the theory of planned behavior (TPB) [26], posit that individual intention is the most direct and strongest determinant of behavior, and researchers usually take this association for granted [27]. However, a behavior–intention gap exists, which refers to the discrepancy between what people claim and how they behave [28,29]. To illustrate, situational factors (e.g., the COVID-19 pandemic) may cause the behavior–intention gap in terms of using O2OFD services [15]. In addition, there are other factors (e.g., habit and facilitation conditions in the UTAUT2) that may predict consumers' actual behavior, which is poorly understood in the context of O2OFD. Thus, the specific objective of this study is to adapt the UTAUT2 to understand consumers' continued intention to use O2OFD services and their subsequent use behavior.

Despite the great convenience of O2OFD services for consumers, they may be related to some negative health problems, such as sedentary behavior (i.e., a sedentary lifestyle), which is a worthy issue regarding consumer well-being. A sedentary lifestyle has been recognized to be associated with multiple adverse health outcomes [[30], [31], [32], [33]], of which sitting time is a common measure. A survey showed that the average worldwide person sits down for 4.7 hours per day [34], and this number may be increasing yearly [35]. Similarly, delivered or take-out food is considered mostly unhealthy [36,37]. During the COVID-19 pandemic, sedentary behavior and the use of O2OFD services were both observed to increase in some way [38]. Possible causes include an increase in work-at-home or study-at-home activities and a decrease in social or outdoor activities. Sedentary lifestyle and the use of O2OFD services are common among members of various organizations who are busy working or studying. Researchers have argued that sedentary lifestyle may be related to the frequent use of O2OFD services [1,39]. Nonetheless, to date, few empirical studies have attempted to explore their relationships. Accordingly, another objective of this study is to explore the direct and indirect effects of sedentary lifestyle on consumers' use of O2OFD services.

To summarize, the primary objective of this study is to delve into consumers' intention to continue using O2OFD services and their consequent usage behavior in the post-pandemic era from the perspective of consumer technology use, investigating specific influencing factors. Additionally, this study aims to explore how sedentary lifestyle influences consumer behavior in using O2OFD services from a consumer health perspective.

## 2 Literature review and research model

### 2.1 Theoretical foundations

#### 2.1.1 Unified theory of acceptance and use of technology 2 (UTAUT2)

The UTAUT2 formulated by Venkatesh et al. [40] was developed based on the unified theory of acceptance and use of technology (UTAUT) [41], which is a social psychology-based technology acceptance theory that synthesizes eight prevailing models ever used to explain the adoption of information systems or technology. The UTAUT2 posits that seven key constructs (i.e., performance expectancy, effort expectancy, social influence, hedonic motivation, price value, facilitating conditions, and habit) directly determine the intention of consumers to use a particular information system and that intention, facilitating conditions, and habit are direct predictors of use behavior. In addition, gender, age, and experience were posited to moderate the relationships in the model. The UTAUT2 is considered the "most comprehensive theory in understanding individual technology adoption and use" [42].

According to the earlier discussion, social psychology-based theories of technology acceptance and use are relevant to understanding consumer behavior in using O2OFD services. Therefore, the UTAUT2 was employed as the underpinning theory in this study. First, the UTAUT2 focuses on technology usage in the consumer context rather than primarily in the organizational setting as its predecessor did. Second, the UTAUT2 aims to explain consumers' intention to use certain information systems and their subsequent use behavior, which is consistent with the objectives of this study. Lastly, Venkatesh et al. [43] proposed a baseline model of the UTAUT/UTAUT2, where the first five original constructs were grouped into individual beliefs and three original moderators were removed, and suggested that researchers make necessary adaptations to the original UTAUT2 model according to their purposes, such as replacing unnecessary individual beliefs. Accordingly, this study adopted the UTAUT/UTAUT2 baseline model and introduced two new individual beliefs (see Section 2.1.2) rather than focusing on the original five.

#### 2.1.2 Theory of network externalities (TNE)

The theory of network externalities (TNE) originated in the domain of economics and was advanced by Katz and Shapiro [44]. Network externalities, also called network effects, refer to the value or utility that a user derives from a product or service depending on the user size of the compatible products or services [44]. According to the TNE, network externalities can be direct or indirect. The former is based on the user size of a given network, whereas the latter is based on the number and availability of complementary products or services. Network externalities are usually positive, which means that as the number of users and complementary products (or services) increases, a given user derives more value from the network.

Although the TNE is an economic theory usually used to analyze macro issues, many researchers have applied it to information systems research at the individual level and verified the effect of network externalities on user behavior (e.g., Refs. [[45], [46], [47]]). In addition, the TNE helps understand the effects of new changes related to the O2OFD on consumer behavior, such as the increase in user size (direct network externality) and the emergence of new products or services (indirect network externality). However, the application of TNE at the micro level should be based on psychological mechanisms, and therefore the perceived network size (i.e., referent network size) and perceived complementarity [45] were introduced into this study to represent direct and indirect network externalities, respectively. In this way, the TNE was used in this study as a supporting theory to explain the effects of perceived network size and perceived complementarity on consumers' continued intention to use O2OFD services in the post-pandemic era.

### 2.2 Hypothesis development

#### 2.2.1 UTAUT2 relationships

Following Venkatesh et al.'s [43] suggestion, this study focused on the predictors of interest rather than having the obligation to replicate the original UTAUT2 model. Specifically, habit and facilitating conditions were selected for this study because they are common predictors for both intention and behavior.

Habit is defined as "the extent to which people tend to perform behaviors automatically because of learning" [40]. Some habit changes caused by the COVID-19 pandemic, such as the use of O2OFD services, may continue in the post-pandemic era [25]. According to the UTAUT2, habit or habitual use of an information system is a predictor of continued use intention and subsequent use behavior. Repeated performance of behavior can form well-developed attitudes and intentions, which in turn affect future behavior [48]. Additionally, use behavior can also be automatically triggered by past use habits without the involvement of intention [49]. Consumers who use O2OFD services frequently during the pandemic may result in usage habits. They may have a stronger continued intention to use O2OFD services and are more likely to continue using these services in the post-pandemic era. Previous O2OFD studies have demonstrated the positive effect of habit on continued intention [4,25]. The direct positive effect of habit on use behavior has also been confirmed in the contexts of mobile banking and mobile shopping [50,51]. Consequently, the following hypotheses are proposed:

H1 Habit (HT) positively influences consumers' continued intention (CI) to use O2OFD services in the post-pandemic era.

H5 Habit (HT) positively influences consumers' use behavior (UB) towards O2OFD services in the post-pandemic era.

Facilitating conditions are defined as "consumers' perceptions of the resources and support available to perform a behavior" [40]. O2OFD services are usually based on mobile applications, involving many information technologies, such as mobile Internet, positioning, and online payment. If a consumer has a set of favorable facilitating conditions that enable them to easily use the O2OFD service, they are more likely to continue using it. The UTAUT2 states that the effect of facilitating conditions on intention is similar to the perceived behavioral control of the TPB [26]. The positive relationship between facilitating conditions and information technology use intention has been demonstrated in studies from different fields [22,50,52]. The UTAUT2 also proposes that facilitating conditions can serve as a proxy for actual behavioral control [26] to directly affect use behavior. Recent information technology studies have further confirmed the positive relationship between facilitating conditions and use behavior [17,23,51]. Accordingly, the following hypotheses are proposed:

H2 Facilitating conditions (FC) positively influence consumers' continued intention (CI) to use O2OFD services in the post-pandemic era.

H6 Facilitating conditions (FC) positively influence consumers' use behavior (UB) towards O2OFD services in the post-pandemic era.

Furthermore, according to the literature, intention is the primary predictor of behavior [40,41]. Therefore, this study hypothesizes that:

H7 Continued intention (CI) positively influences consumers' use behavior (UB) towards O2OFD services in the post-pandemic era.

#### 2.2.2 Network externalities

The perceived network size represents the direct network externality, which refers to the consumer's perception of the number of O2OFD users based on their social circle. Lin and Bhattacherjee [45] pointed out that the network benefits users obtain from a particular information system depend not on the number of actual total users but on those in their social circle. From a psychological perspective, the user size of O2OFD perceived by consumers is also largely referenced to their social circles. As a growing number of friends or acquaintances begin to use a particular information system, its attractiveness to given users will increase [45]. According to the TNE, when the network size reaches a critical point, a bandwagon effect can occur. Therefore, it can be expected that the increase in network size due to the pandemic may cause consumers to continue using O2OFD services in the post-pandemic era. In addition, perceived network size can be regarded as replacing social influence in the UTAUT2, which refers to "the extent to which consumers perceive that important others believe they should use a particular technology" [40].

Perceived complementarity represents the indirect network externality, which refers to the consumer's perception of the availability of complementary products or services on the O2OFD platform. According to the TNE, the benefits obtained by users from a particular network increase with the variety of complementary products or services available, which in turn attracts more users. Similar to network size, the use of a certain information system by given users depends on their perception of the availability of complementary products or services rather than on actual availability [45]. Previous studies have confirmed that perceived complementarity can contribute to perceived usefulness (i.e., performance expectancy) [53,54], and hence perceived complementarity can be considered as one of the dimensions of performance expectancy in the UTAUT2. Accordingly, the more variety of products or services an O2OFD platform offers, the more useful consumers will think it is, and the more likely they are to continue using the O2OFD service in the post-pandemic era.

From the perspective of psychology, network externalities are perceived by the consumer, which in this study were viewed as individual beliefs in the UTAUT2 directly and positively influencing consumers' continued intention to use O2OFD services. Empirical studies in different contexts have shown that perceived network size and perceived complementarity directly or indirectly influence users' intention to use a particular information system [46,47,55]. Therefore, the following hypotheses are proposed:

H3 Perceived network size (PNS) positively influences consumers' continued intention (CI) to use O2OFD services in the post-pandemic era.

H4 Perceived complementarity (PC) positively influences consumers' continued intention (CI) to use O2OFD services in the post-pandemic era.

#### 2.2.3 Roles of sedentary lifestyle

Sedentary behavior (i.e., a sedentary lifestyle) refers to "any waking behavior characterized by an energy expenditure ≤1.5 metabolic equivalents (METs), while in a sitting, reclining or lying posture" [30]. To date, no studies have demonstrated the relationship between consumers' use of O2OFD services and their sedentary lifestyles, and thus the study on the roles of sedentary lifestyle in the context of O2OFD was exploratory.

Sedentary time is a common measure of sedentary lifestyle [34], and a highly sedentary individual generally has relatively less time for other activities, such as preparing food. Busyness (lack of time) and laziness are factors related to sedentary lifestyle [32]. People who are either busy or lazy are more likely to use O2OFD services frequently because they may not have the time or inclination to prepare food by themselves. Studies have shown that time-saving or convenience-seeking is one of the motivations for consumers to adopt O2OFD services [18,22]. In addition, negative self-perception of health is another factor associated with sedentary lifestyle [32]. Similarly, consumers who frequently use O2OFD services are generally not concerned with the nutritional and health aspects of the delivered food [56]. At this point, highly sedentary individuals are likely to be frequent users of O2OFD services. Although the causal relationship is currently unclear, sedentary lifestyle may be associated with O2OFD service usage. Therefore, this study hypothesized that:

H8 *Sedentary lifestyle (SL) is positively related to consumers' use behavior (UB) towards O2OFD services*.

Furthermore, according to the UTAUT2, the relationships in the model may vary from individual differences [40]. Accordingly, sedentary lifestyle may moderate the effects of habit, facilitating conditions, and intention on the use of O2OFD services. The habit–behavior association is established in stable environments, where behavior can be triggered by familiar stimulus cues [40]. If consumers perceive the changing environment as relatively unstable, the link between habit and behavior can easily be broken. Highly sedentary individuals perceive the environment as relatively stable because they maintain the same lifestyle or state for a long time, and thus habit has a stronger effect on their use of O2OFD services. Highly sedentary individuals typically rely more on and use more frequently O2OFD services. As the experience of using O2OFD services increases, their routine behavior becomes automatic without control of intention, and the habit is reinforced [40]. As a result, the connection between intention and the use of O2OFD services is weakened for highly sedentary consumers. In addition, sedentary lifestyle involves prolonged use of digital products, such as computers or mobile devices [35,57,58]. It can be assumed that highly sedentary consumers are more familiar with information technology and hence rely less on facilitating conditions when using O2OFD services. Therefore, the following hypotheses are suggested:

H8a Sedentary lifestyle (SL) strengthens the effect of habit (HT) on consumers' use behavior (UB) towards O2OFD services.

H8b Sedentary lifestyle (SL) weakens the effect of facilitating conditions (FC) on consumers' use behavior (UB) towards O2OFD services.

H8c Sedentary lifestyle (SL) weakens the effect of continued intention (CI) on consumers' use behavior (UB) towards O2OFD services.

**Fig. 1:**
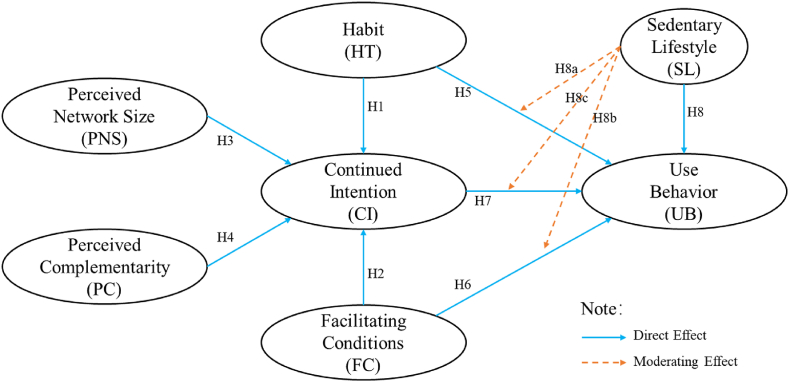
Research framework.

Based on the discussion, a research framework is proposed, as shown in Fig. 1.

## 3 Methodology

### 3.1 Research design

Conducting research on human behavior generally requires selecting a research paradigm to improve credibility and generalizability. Based on the purpose of predicting consumer behavior, this study adopted the positivism paradigm, which emphasizes that objective data can be used to assess relationships between factors and to test hypotheses or theories. Following the positivist paradigm, a quantitative method with a survey strategy was employed. Specifically, theories were employed in the study to explain the relationships between factors and to propose hypotheses. Subsequently, primary data were collected through a survey, and the hypotheses were tested using statistical analysis. This study is an extension of a dissertation, which was approved by the ethics committee of Universiti Putra Malaysia (JKEUPM-2022-463). Digital informed consent was obtained online from all subjects involved in the study.

### 3.2 Research instruments

In this study, the research instruments were developed based on the existing literature, as shown in Table 1. Predictor variables (i.e., habit, facilitating conditions, perceived network size, and perceived complementarity) were measured respectively using a five-point Likert scale, while continued intention used a seven-point Likert scale. The ultimate outcome variable use behavior was measured using a single item, anchored from 1 = "once every 4 months (or longer)" to 9 = "once a day." Although use behavior may be a broader concept, researchers have suggested that constructs consisting of a singular concrete object and concrete attribute should use a single-item measure in marketing research [59]. Regarding the moderating variable sedentary lifestyle (i.e., sedentary time), the total assessment (i.e., single-item questionnaire) based on self-report, such as the international physical activity questionnaire (IPAQ) [60], is commonly used [34]. Accordingly, a single item adapted from the IPAQ was used to measure sedentary time, anchored from 1 = "4 hours a day or less" to 7 = "10 hours a day or more." In addition, this study used different scales, which was suggested as one of the procedural remedies to reduce common method bias (CMB) [61].

**Table 1:** Measurement of variables.

Variable	Item	Adapted from
Habit (HT)	HT1: The use of O2OFD services has become a habit for me.	Venkatesh et al. [40]
	HT2: I must use O2OFD services in my daily life.	
	HT3: When I want to buy food for a meal, using the O2OFD service is an obvious choice for me.	
Facilitating conditions (FC)	FC1: I have the resources necessary to use O2OFD services.	Venkatesh et al. [40]
	FC2: I have the knowledge necessary to use O2OFD services.	
	FC3: O2OFD services are compatible with other technology I use.	
Perceived network size (PNS)	PNS1: What percentage of your peers (in work or school) use the O2OFD service?	Lin and Bhattacherjee [45]
	PNS2: What percentage of your friends use the O2OFD service?	
	PNS3: What percentage of your personal circle uses the O2OFD service?	
Perceived complementarity (PC)	PC1: A wide range of products is available on O2OFD platforms.	Lin and Bhattacherjee [45]
	PC2: A wide range of services is available on O2OFD platforms.	
	PC3: A wide range of functions is available on O2OFD platforms.	
Continued intention (CI)	CI1: I intend to continue using O2OFD services after the pandemic recedes.	Venkatesh et al. [40]
	CI2: Even if the pandemic subsides, I will always try to use O2OFD services.	
	CI3: I will continue to use O2OFD services frequently in the post-pandemic era.	
Use behavior (UB)	Please choose your frequency for using the O2OFD service.	Venkatesh et al. [40]
Sedentary lifestyle (SL)	During the past period, how many hours per day on average did you remain sitting, reclining, or lying stationary while waking?	Craig et al. [60]

### 3.3 Sampling and data collection

The survey was conducted on China's mainland. This is because China is the leading country in the O2OFD industry and has experienced a surge in user size during the pandemic [16], where respondents relevant to the study were easy to recruit. Since the sampling frame was unavailable, this study adopted a non-probability sampling approach. Specifically, a purposive sampling technique was employed, and the inclusion criteria for respondents were that (1) they must have been at least 18 years of age, (2) they must have ever used the O2OFD service due to being impacted by the pandemic, and (3) they must have used the O2OFD service at least once in the last six months. These criteria can ensure that the sample provides information relevant to the study while minimizing sampling error. Data were collected online in two stages, where the data for key predictors and continued intention were collected in the first stage, while data for use behavior and sedentary lifestyle were collected in the second stage (approximately two months later). Using a two-stage survey was another procedural remedy to reduce the CMB [61].

According to Sekaran and Bougie [62], sample sizes greater than 30 and less than 500 are appropriate for most studies. Given the attrition rate of respondents and the unavailability of responses, a sample size of 600 was adopted for this study. The survey was completely anonymous and voluntary, with data collected through an online platform. A total of 600 responses were received in November 2022 (the first stage), of which 425 respondents participated in the second stage survey in January–February 2023. After excluding invalid responses, 409 responses were ultimately used in the subsequent data analysis. The profile of the respondents is shown in Table 2.

**Table 2:** The profile of respondents (N = 409).

Field	Category	Frequency	Percentage (%)
Gender	Male	181	44.25
	Female	228	55.75
Age (years old)	18–25	151	36.92
	26–35	165	40.34
	36–41	72	17.60
	42 and above	21	5.14
Education	Diploma and below	55	13.45
	Undergraduate	257	62.84
	Postgraduate	97	23.72
Occupation	Student	87	21.27
	Manager	101	24.69
	Non-manager	198	48.41
	Self-employed	18	4.40
	Other	5	1.22

### 3.4 Data analysis

This study primarily used structural equation modeling (SEM) for data analysis, which enables researchers "to simultaneously model and estimate complex relationships among multiple dependent and independent variables" [63]. Specifically, partial least squares structural equation modeling (PLS-SEM) was applied in this study to test the hypotheses. The PLS-SEM has become a common technique in marketing research and is suitable for evaluating complex relationships (e.g., moderation) and for exploratory studies [64]. In addition, PLS-SEM proves to be valuable when using single-item measurements because this method does not suffer from identification problems [63]. The SmartPLS 3.3.9 software was employed to perform the PLS-SEM.

## 4 Results

### 4.1 Data examination

Before formal analysis, the data were examined to ensure that they met the requirements. First, since the data were collected digitally, missing values and outliers were almost impossible to exist. Second, although PLS-SEM makes no distributional assumptions [65], extremely non-normal data are considered problematic. This study examined the kurtosis and skewness of all parameters, and they were all between −2 and +2, respectively, indicating that the data distribution was acceptable [63]. Third, the CMB should not be a serious concern in this study because a set of procedural remedies have been applied to reduce it. The full collinearity method [66] was used to detect the potential CMB, and the variance inflation factor (VIF) values for all variables were below the threshold value of 3.33 [67], confirming that the CMB was not a serious problem.

### 4.2 Assessment of measurement models

All constructs in this study adopted reflective measurements, and the assessment of measurement models involved assessing the reliability (i.e., indicator and internal consistency reliability) and validity (i.e., convergent and discriminant validity). The indicator's outer loading was used to examine the indicator reliability, and it should be greater than 0.70 for each indicator [63]. The reliability coefficient (Rho_A) is considered a good representation of a construct's internal consistency, which is usually between the values of Cronbach's alpha (Alpha) and composite reliability (CR) and should generally be greater than 0.70 but less than 0.95 [63]. The average variance extracted (AVE) was used to assess the convergent validity, which should be higher than 0.50 for each construct [63]. The heterotrait-monotrait (HTMT) ratio was adopted to evaluate the discriminant validity, and it should be less than 0.85 for conceptually different constructs [68]. Table 3 and Table 4 summarize the results of the measurement model assessment, where all evaluation criteria were met, supporting the measures' reliability and validity in the current study.

**Table 3:** Reliability of measures.

Variable	Item	Loading	Alpha	Rho_A	CR
HT	HT1	0.821	0.752	0.755	0.859
	HT2	0.854			
	HT3	0.778			
FC	FC1	0.824	0.736	0.742	0.850
	FC2	0.777			
	FC3	0.823			
PNS	PNS1	0.888	0.848	0.859	0.908
	PNS2	0.847			
	PNS3	0.890			
PC	PC1	0.815	0.727	0.729	0.846
	PC2	0.810			
	PC3	0.786			
CI	CI1	0.832	0.824	0.827	0.895
	CI2	0.871			
	CI3	0.876

**Table 4:** Validity of measures.

Variable	HT	FC	PNS	PC	CI	UB	SL
HT	**0.670**						
FC	0.333	**0.654**					
PNS	0.488	0.379	**0.766**				
PC	0.614	0.424	0.365	**0.646**			
CI	0.761	0.438	0.541	0.699	**0.739**		
UB	0.736	0.407	0.564	0.598	0.738	**1.000**	
SL	0.442	0.304	0.234	0.204	0.216	0.359	**1.000**

Note: AVE values are displayed on the diagonal; the confidence interval upper bounds of the HTMT ratio are contained in the lower left triangle (bootstrapping with 10,000 sub-samples, α = 0.05).

### 4.3 Assessment of structural models

The assessment of structural models primarily included evaluating the collinearity issues, significance of path coefficients, and explanatory power. The assessment results are summarized in Table 5. First, all VIF values were less than the threshold value of 3.00, suggesting that collinearity among the exogenous variables was not a critical issue in each sub-model [63]. Second, all the path coefficients were significant, and therefore H1–8 were supported. However, the effect size (ƒ^2^) values of both FC–CI and FC–UB were less than 0.02, which were considered as having no measurable effect. Third, the coefficient of determination (R^2^) values of the endogenous constructs (i.e., CI and UB) were 0.436 and 0.493, respectively, which represented the explanatory power of the structural model. Compared with the original UTAUT2 model [40], the explanatory power of the model in this study was acceptable.

**Table 5:** Assessment results of structural models.

Hypothesis: Path	VIF	Path coefficient	p-value	Confidence interval	ƒ^2^	R^2^
H1: HT–CI	1.273	0.356 **	<0.001	[0.287, 0.420]	0.177	0.436 (CI)
H2: FC–CI	1.096	0.097 **	0.007	[0.036, 0.164]	0.015	
H3: PNS–CI	1.164	0.200 **	<0.001	[0.139, 0.263]	0.061	
H4: PC–CI	1.223	0.281 **	<0.001	[0.216, 0.343]	0.114	
H5: HT–UB	1.569	0.300 **	<0.001	[0.228, 0.372]	0.113	0.493 (UB)
H6: FC–UB	1.103	0.087 **	0.011	[0.025, 0.151]	0.013	
H7: CI–UB	1.507	0.417 **	<0.001	[0.344, 0.484]	0.228	
H8: SL–UB	1.143	0.126 **	<0.001	[0.070, 0.184]	0.028	

Note: ** significant (bootstrapping with 10,000 sub-samples, α=0.05).

### 4.4 Moderation analysis

**Fig. 2:**
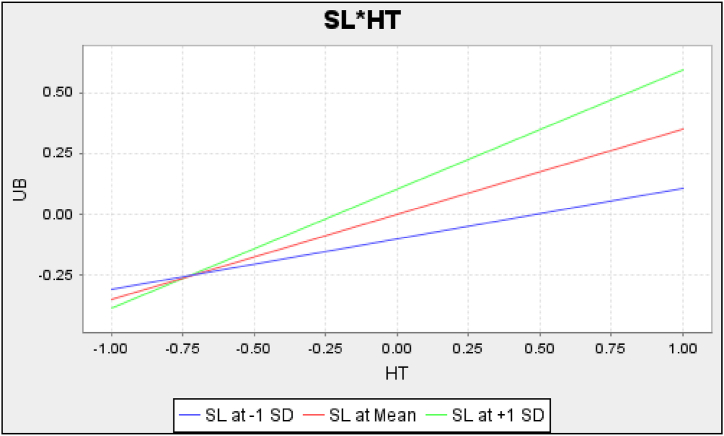
The moderating effect of SL on HT.

**Fig. 3:**
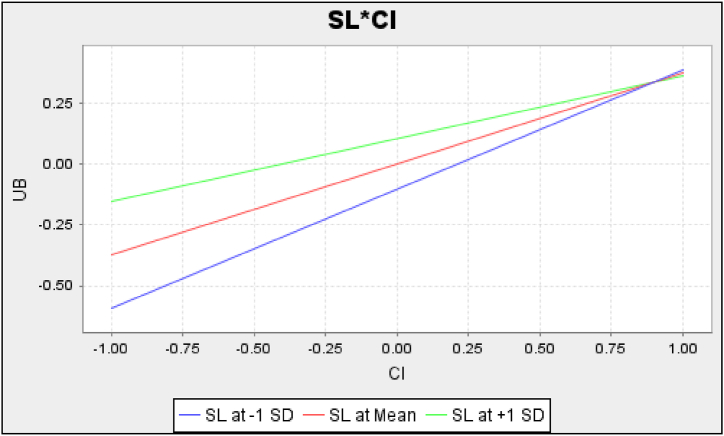
The moderating effect of SL on CI.

To analyze the moderating effects of sedentary lifestyle (SL), the two-stage approach [69] was used to create interaction terms (i.e., SL*HT, SL*CI, and SL*FC). Subsequently, the sub-model of UB with the addition of interaction terms was assessed, and the results are shown in Table 6. The path coefficient of SL*HT–UB was positive and significant, and therefore H8a was supported. The path coefficient of SL*CI–UB was negative and significant, and thus H8c was supported. The ƒ^2^ values of SL*HT–UB and SL*CI–UB were 0.023 and 0.016, respectively, which were considered medium effects in the moderation analysis [63]. However, the relationship SL*FC–UB was non-significant, and hence H8b was not supported. The significant moderating effects are further demonstrated in Fig. 2, Fig. 3. In addition, the R^2^ value of UB increased from 0.493 to 0.508 owing to the inclusion of the moderating effects.

**Table 6:** Assessment results of the UB structural model with interaction terms.

Hypothesis: Path	Path coefficient	p-value	Confidence interval	ƒ^2^	R^2^
H5: HT–UB	0.349 **	<0.001	[0.273, 0.423]	0.143	0.508
H6: FC–UB	0.074 **	0.024	[0.013, 0.136]	0.010	
H7: CI–UB	0.373 **	<0.001	[0.297, 0.442]	0.174	
H8: SL–UB	0.103 **	0.003	[0.042, 0.164]	0.018	
H8a: SL*HT–UB	0.141 **	<0.001	[0.072, 0.21]	0.023	
H8b: SL*FC–UB	−0.051	0.060	[-0.107, 0.002]	0.005	
H8c: SL*CI–UB	−0.116 **	0.002	[-0.179, −0.048]	0.016	

Note: ** significant (bootstrapping with 10,000 sub-samples, α=0.05).

## 5 Discussion

This study aimed to investigate the factors influencing consumers' continued intention to use O2OFD services and their subsequent use behavior in the post-pandemic era, as well as to explore the roles of sedentary lifestyle. A research model based on the UTAUT2, integrating network externalities and sedentary lifestyle, was proposed. An online survey was conducted in China, and the PLS-SEM was applied to test the proposed hypotheses.

### 5.1 Discussion of results

In this study, the UTAUT/UTAUT2 baseline model proposed by Venkatesh et al. [43] was adopted, in which individual beliefs, habit, and facilitating are theorized to predict behavioral intention, while habit, facilitating conditions, and behavioral intention to determine use behavior. This study modified the baseline model according to the current context. Specifically, individual beliefs were represented by two network externality factors, and sedentary lifestyle was added to the research model as a contextual factor to influence use behavior. The study model was empirically tested, with all direct effects being significant (H1-8 being supported), indicating that the theory extension obtained the expected effectiveness in the current context. The corresponding predictors in the structural model explained 43.6% and 49.3% of the variance in continued intention (R^2^ = 0.436) and use behavior (R^2^ = 0.493, without moderating effects), respectively. The R^2^ value represents the exploratory power of the research model, which usually grows with the number of predictors in the model and should be interpreted based on similar models in relevant studies [63]. By comparing with the original model and similar studies [17,40,50,52], the exploratory power of the current research model is considered acceptable. This, in turn, demonstrates the theoretical foundation of the current study.

The results show that habit significantly and positively affected consumers' continued intention, indicating that consumers who form habitual behavior regarding the use of O2OFD services are more likely to have a higher intention to continue using them in the post-pandemic era. Habit was the strongest predictor in terms of the path coefficient, which is largely consistent with previous studies on O2OFD services [4,24,25]. A meta-analysis also showed that habit was the strongest predictor of behavioral intention in UTAUT2 [42]. The intention will, in turn, influence the future behavior of consumers. However, when a habit is formed, behavior can also be activated directly by stimulus cues without the involvement of intention [40]. This is confirmed by the present study, with habit significantly and positively influencing consumer behavior in using O2OFD services. Several studies have presented similar findings in different contexts, such as live-streaming e-commerce [70], health information applications [71], and lecture capture systems [72].

The results also show that perceived complementarity and perceived network size significantly and positively influenced consumers' continued intention, suggesting that positive network externalities would encourage consumers to continue using O2OFD services in the post-pandemic era. Perceived complementarity was the second strongest predictor of continued intention in this study. It is regarded as related to perceived usefulness (i.e., performance expectancy in the UTAUT2). Previous studies have shown that the effect of perceived complementarity on intention might be non-significant when mediators (e.g., trust or perceived usefulness) were present in the model [46,73]; otherwise, it could effectively predict intention [47,55], as demonstrated in the current study. In addition, previous studies have indicated that perceived network size positively influences consumers' continued intention or behavior [46,73], which is consistent with the current study. The role of perceived network size can also be explained by herd behavior [74], which suggests that individuals usually choose the same actions as the majority of those around them. This study argued that perceived network size is similar to social influence in the UTAUT2. Interestingly, some studies have reported non-significant effects of social influence on continued intention [23,75]. In fact, although most information technology studies found the relationship between social influence and behavioral intention as significant, they reported minimal path values [42]. One possible explanation is that conceptualizing social influence as the opinions of important others is not suitable for the contexts of most underlying technologies. Therefore, this study concludes that perceived network size is more suitable in the current context.

Regarding facilitating conditions, although the effect on continued intention was significant and consistent with the earlier prediction, the effect size (ƒ^2^ = 0.015, see Table 5) was regarded as trivial, implying that facilitating conditions may not be a key predictor in the current context. One possible reason for this is that this study focused on continued usage; therefore, the respondents were all experienced users. According to Venkatesh et al. [40], the effect of facilitating conditions on intention diminishes with increasing experience. In fact, several O2OFD studies focusing on continued intention have yielded non-significant effects of facilitating conditions [4,24,25]. Similarly, the effect of facilitating conditions on use behavior was significant, but the effect size (ƒ^2^ = 0.013, see Table 5) was trivial, which is in line with a recent study on health information applications [71]. Overall, the significant path coefficients support the theoretical foundation of this study, but the trivial effect sizes suggest that facilitating conditions may not warrant managerial attention in the current context.

Consistent with the majority of theories and studies (e.g., Refs. [26,40,71,72]), intention was the primary determinant of behavior in this study. Nevertheless, a behavior–intention gap exists, and situational factors may lead to a discrepancy between intention and behavior [15,28,29]. Furthermore, consumers' use of O2OFD services in the post-pandemic era may also be determined or influenced by other factors, such as habit and sedentary lifestyle.

This study also aimed to explore the roles of sedentary lifestyle in consumers' use of O2OFD services. The findings indicate that sedentary lifestyle was significantly and positively related to use behavior, suggesting that high-sedentary consumers would use O2OFD services more frequently. As discussed earlier, busyness, laziness, or negative self-perception of health may be the reason for the frequent use of O2OFD services by high-sedentary consumers. In addition, a study has shown that high-sedentary individuals have stronger brain responses to food cues after caloric intake compared to active individuals [76]. This means that high-sedentary consumers are more likely to use O2OFD services to order tea-time items and night snacks.

Furthermore, the findings demonstrate the moderating roles of sedentary lifestyle. First, the habit–behavior relationship was moderated by sedentary lifestyle in the context of O2OFD, where the effect of habit was stronger for high-sedentary consumers, as shown in Fig. 2. The explanation for this moderating effect on habit is based on the assumption that the changing environment is relatively stable for high-sedentary consumers. The formation and triggering of a habit are related to the cues in the environment of using O2OFD services; the link between stimulus cues and O2OFD use behavior is established in a relatively stable environment [40]. High-sedentary consumers perceive the environment as relatively stable because they are sedentary for long hours and do not have the time or willingness to prepare food themselves, and therefore the association between habit and behavior is more easily established and reinforced. Conversely, low-sedentary consumers have more options and considerations in terms of food preparation or purchase, and thus the changing environment may disrupt the habit–behavior association, making their behavior uncontrolled by habit. Second, the effect of continued intention on subsequent O2OFD use behavior was moderated by sedentary lifestyle. As shown in Fig. 3, the relationship between continued intention and use behavior was stronger for low-sedentary consumers. The explanation for this moderating effect is based on the assumption that high-sedentary consumers rely more on O2OFD services. Specifically, high-sedentary consumers use O2OFD services more frequently, resulting in their use behavior becoming automated and more guided by habits (cues) rather than controlled by their intention [40]. Thus, the effect of intention on high-sedentary consumers would be weaker than on low-sedentary ones. This is also an explanation of the behavior–intention gap. Third, sedentary lifestyle did not moderate the effect of facilitating conditions on use behavior in the current context. One explanation might be that the O2OFD industry has already been very popular in the study site, and hence the between-group difference in facilitation conditions may not exist or may be difficult to detect.

### 5.2 Theoretical implications

This study theoretically adds value to knowledge in three ways. First, the UTAUT2 was modified and extended to the current research context, which is regarded as a form of novelty [77]. Specifically, the baseline model of UTAUT2 [43] was extended to explain consumer behavior in the context of O2OFD. Although the UTAUT2 from the information technology field has been extensively validated in various contexts, the developers of the theory recommended re-conceptualizing the use of technology to refine the current context effect [43]. Additionally, most studies have focused only on explaining consumers' intention rather than actual behavior. In response, the present study provides empirical evidence for the application of the UTAUT2 baseline model in the O2O commerce context and provides theoretical insights for understanding consumers' use of O2OFD services.

Second, the theory of network externalities [44] from the domain of economics was integrated into the research model to provide a theoretical lens for understanding consumers' continued use of O2OFD services in the post-pandemic era. The user size of O2O services, especially O2OFD services, surged during the pandemic, which has, in a sense, promoted innovation in business models, with many new or complementary services on O2OFD platforms emerging or becoming popular. Network externalities were hence applied at the individual level to explain the effects of perceived network size and perceived complementarity on consumers' continued intention from a psychological perspective. Such integration is considered a key contribution and a way to advance science [78].

Third, this study adds value to the existing consumer literature by exploring the roles of sedentary lifestyle. A sedentary lifestyle is directly related to the physical well-being of consumers [32,33], which is an important aspect of consumer well-being. However, the roles of sedentary lifestyle in consumer behavior are largely unknown. Therefore, this study examined the relationship between sedentary lifestyle and the use of O2OFD services, as well as explored the moderation effects of sedentary lifestyle to identify the boundary condition of theory, offering interesting insights into consumer behavior.

### 5.3 Practical implications

This study also offers practical implications for many aspects. Several marketing strategies may be given to O2OFD platform operators. First, habit is one of the primary determinants of consumers' continued use of O2OFD services. Therefore, marketers should develop appropriate promotions to enable prospective or low-frequency users to experience services that may prompt habit formation while avoiding significant changes in business processes and user interfaces that may disrupt the habits of existing customers. Second, perceived network size, rather than actual user size, can influence consumers' continued intention. Thus, marketers may adopt social-based promotion strategies to increase consumers' perceived network size. Third, the designer of business models or platforms can develop a wide range of complementary services or tools to encourage consumers to use O2OFD applications. For example, O2OFD platforms in China commonly offer a variety of services, such as medicine, flowers, and fresh food delivery.

On the other hand, consumers should understand their behavior when using O2OFD services to make more informed decisions. First, consumers should recognize that their use of O2OFD services may be habitual, which may lead to over-ordering and uninformed purchases. Second, they should be aware that the frequent use of O2OFD services may be related to negative lifestyles, such as unhealthy diets and sedentary behavior. In addition, consumers, especially those who use O2OFD services frequently, should self-assess whether their sedentary time is too high and take appropriate initiatives.

Moreover, this study provides valuable information to policymakers. Governments should realize that digitalization is leading to transformations in the food industry and changes in consumer behavior, which should be taken into account when developing policies. Business and economic policymakers may use the findings of this study to develop strategies to encourage the sustainable development of the O2OFD industry, further ensuring job opportunities for a large number of delivery workers to reduce unemployment, as well as contributing to social vigor and economic growth. In addition, public health policymakers should make efforts to increase consumers' health awareness in terms of their daily diet and physical activity. They can also develop interventions or laws to reduce the negative impact arising from the use of O2OFD services.

### 5.4 Limitations and future directions

Several limitations need to be noted regarding the current study, and they can be considered worthy directions for future research. First, this study focused on a specific country (i.e., China), which implies that the generalization of the findings may be restricted. Given the global popularity of O2OFD services, future research is recommended to use cross-country samples to deepen the understanding of O2OFD continuance. Second, this study used a subjective (self-report) approach to measure sedentary lifestyle, which may not reflect the actual daily sedentary time of the respondents. Therefore, findings related to sedentary lifestyle should be interpreted cautiously, and future research is suggested to develop better subjective measures to study consumers' sedentary lifestyles. Third, the causal relationship between sedentary lifestyle and O2OFD service usage is unclear, and future research is thus recommended to adopt an experimental design to further understand their relationship.

## 6 Conclusion

The COVID-19 pandemic has changed consumer behavior and habits in many ways. For example, many people have begun or increased their use of various online-to-offline (O2O) services, including the O2O food delivery (O2OFD) service. As the post-pandemic era begins, these habits may continue or disappear. Therefore, this study set out to investigate consumers' continued use of O2OFD services in the post-pandemic era. The findings indicate that habit was the primary predictor of consumers' continued intention to use O2OFD services, as well as that an increase in user size (i.e., perceived network size) and the emergence of new services (i.e., perceived complementarity) had a significant positive effect on continued intention. Habit and continued intention were the main determinants of consumers' use behavior; however, their effects were moderated by sedentary lifestyle. During the pandemic, people's sedentary time and O2OFD service usage were observed to increase, which may be related to adverse health outcomes. To date, there is little understanding of the relationship between sedentary lifestyle and O2OFD service usage in consumer research. Thus, another purpose of this study is to explore the roles of sedentary lifestyle in consumers' use of O2OFD services. The results show that sedentary lifestyle was positively related to the use behavior towards O2OFD services. Additionally, sedentary lifestyle strengthened the effect of habit and weakened the effect of continued intention on use behavior. Despite some limitations, this study brings theoretical and practical implications for understanding consumers' continued use of O2OFD services in the post-pandemic era and offers interesting insights into consumer well-being by exploring the roles of sedentary lifestyle.

## Declarations

### Author contribution statement

Pinyi Yao: Conceived and designed the experiments; Analyzed and interpreted the data; Wrote the paper.

Syuhaily Osman, Mohamad Fazli Sabri, Norzalina Zainudin: Conceived and designed the experiments; Wrote the paper.

Yezheng Li: Performed the experiments; Contributed reagents, materials, analysis tools or data.

### Data availability statement

Data will be made available on request.

## Declaration of competing interest

The authors declare that they have no known competing financial interests or personal relationships that could have appeared to influence the work reported in this paper.
