# Supplementary material for: Do consumers continue to use O2O food delivery services in the post-pandemic era? Roles of sedentary lifestyle
Source: Heliyon. 2023 Aug 15;9(9):e19131. doi: 10.1016/j.heliyon.2023.e19131 (PMC10472249; doi:10.1016/j.heliyon.2023.e19131)
Supplement: Multimedia component 1 [file mmc1.docx]

**Invitation to Participate in a Questionnaire on:**

**Online-to-Offline Food Delivery (O2OFD) Service**

Dear participant,

The present survey as a part of a dissertation aims to investigate consumers' continued use of the online-to-offline food delivery (O2OFD) service in the post-COVID-19-pandemic era. O2OFD allows offline food providers to expand their business using online channels and brings great convenience and diverse options to diners. Specifically, local restaurants or food outlets usually partner with third-party platforms (e.g., Meituan and Ele.me) to provide instant food delivery, while consumers typically choose and purchase food through a mobile application and subsequently receive the delivery at a location of their preference.

This is a two-stage online survey designed to understand the factors related to consumers' continued use of O2OFD service. Respondents aged 18 or above will be invited to participate in the study. After you complete the first stage of the questionnaire, you will be invited to participate in the second stage of the survey approximately two months later, which takes about 10 minutes each. Your responses to all the questions need only be based on your views and experience, and there is no risk involved in answering any of the questions. Your responses are completely anonymous and will only be published in aggregate form for academic purposes. The information provided will be treated as strictly confidential, and no data about you will be released. Remember that your participation in this survey is voluntary, which means that you are free to participate or not, as well as give up at any time.

Consent

I declare that I am 18 or over 18 years old and agree to participate in this research. I have been informed about the purpose and potential risks of the research. I understand that I have the right to withdraw from this research at any time without giving any reason whatsoever. I also understand that this research is confidential and all information provided with regard to my identity will remain private and confidential.

**Stage 1**

| 1. **Consent** | |
| --- | --- |
| I agree to participate in this survey | I do not agree to participate in this survey |

**Basic Information**

| 2. Have you ever used some form of O2OFD service due to the impact of the COVID-19 pandemic? | |
| --- | --- |
| Yes, permanent employee | No |
|  | |
| 3. Have you used some form of O2OFD service in the last six months? | |
| Yes | No |
|  | |
| 4. Gender | |
| Male | Female |
|  | |
| 5. Age | |
| ________ years old. | |

**Perceptions related to the use of O2OFD service**

| **Habit** | **Strongly Disagree** | **Disagree** | **Neutral** | **Agree** | **Strongly Agree** |
| --- | --- | --- | --- | --- | --- |
| 6. The use of O2OFD services has become a habit for me. | 1 | 2 | 3 | 4 | 5 |
| 7. I must use O2OFD services in my daily life. | 1 | 2 | 3 | 4 | 5 |
| 8. When I want to buy food for a meal, using the O2OFD service is an obvious choice for me. | 1 | 2 | 3 | 4 | 5 |

| **Facilitating conditions** | **Strongly Disagree** | **Disagree** | **Neutral** | **Agree** | **Strongly Agree** |
| --- | --- | --- | --- | --- | --- |
| 9. I have the resources necessary to use O2OFD services. | 1 | 2 | 3 | 4 | 5 |
| 10. I have the knowledge necessary to use O2OFD services. | 1 | 2 | 3 | 4 | 5 |
| 11. O2OFD services are compatible with other technology I use. | 1 | 2 | 3 | 4 | 5 |

| **Perceived network size** | **< 20%** | **20-40%** | **40-60%** | **60-80%** | **> 80%** |
| --- | --- | --- | --- | --- | --- |
| 12. What percentage of your peers (in work or school) use the O2OFD service? | 1 | 2 | 3 | 4 | 5 |
| 13. What percentage of your friends use the O2OFD service? | 1 | 2 | 3 | 4 | 5 |
| 14. What percentage of your personal circle uses the O2OFD service? | 1 | 2 | 3 | 4 | 5 |

| **Perceived complementarity** | **Strongly Disagree** | **Disagree** | **Neutral** | **Agree** | **Strongly Agree** |
| --- | --- | --- | --- | --- | --- |
| 15. A wide range of products is available on O2OFD platforms. | 1 | 2 | 3 | 4 | 5 |
| 16. A wide range of services is available on O2OFD platforms. | 1 | 2 | 3 | 4 | 5 |
| 17. A wide range of functions is available on O2OFD platforms. | 1 | 2 | 3 | 4 | 5 |

| **Continued intention** | **Strongly**  **Disagree** | **Disagree** | **Slightly**  **Disagree** | **Neutral** | **Slightly**  **Agree** | **Agree** | **Strongly**  **Agree** |
| --- | --- | --- | --- | --- | --- | --- | --- |
| 18. I intend to continue using O2OFD services after the pandemic recedes. | 1 | 2 | 3 | 4 | 5 | 6 | 7 |
| 19. Even if the pandemic subsides, I will always try to use O2OFD services. | 1 | 2 | 3 | 4 | 5 | 6 | 7 |
| 20. I will continue to use O2OFD services frequently in the post-pandemic era. | 1 | 2 | 3 | 4 | 5 | 6 | 7 |

**Stage 2**

| 1. **Consent** | |
| --- | --- |
| I agree to participate in this survey | I do not agree to participate in this survey |

**Actual Use**

| 2. Please choose your frequency for using the O2OFD services: |
| --- |
| Once every 4 months (or longer) |
| Once every 2-3 months |
| Once a month |
| Once every 3 weeks |
| Once every 2 weeks |
| Once every 6-7 days |
| Once every 4-5 days |
| Once every 2-3 days |
| Almost once a day |

**Demographic Information**

| 3. Gender | | | |
| --- | --- | --- | --- |
| Male | | Female | |
|  | | | |
| 4. Age (years old) | | | |
| Under 18 | 18-25 | | 26-35 |
| 36-41 | 42 and above | |  |
|  | | | |
| 5. Education | | | |
| Diploma and below | Undergraduate | | Postgraduate |
|  |  | |  |
| 6. Occupation |  | |  |
| Manager | Non-Manager | | Self-employed |
| Student | Orders | |  |

**Sedentary Lifestyle**

| 7. During the past period, how many hours per day on average did you remain sitting, reclining, or lying stationary while waking? |
| --- |
| 4 hours a day (or less) |
| 5 hours a day |
| 6 hours a day |
| 7 hours a day |
| 8 hours a day |
| 9 hours a day |
| 10 hours a day (or more) |

----- END -----
